# Whole-Genome Sequence of an Orf Virus Isolate Derived from a Cell Culture Infected with Contagious Ecthyma Vaccine

**DOI:** 10.1128/MRA.00752-20

**Published:** 2020-08-06

**Authors:** Daniel L. Heare, Sara V. Little, Dale W. Weise, James R. Harris, Andrew E. Hillhouse, Kranti Konganti, Sara D. Lawhon

**Affiliations:** a Department of Veterinary Pathobiology, College of Veterinary Medicine and Biomedical Sciences, Texas A&M University, College Station, Texas, USA; b Texas Vet Lab, Inc., San Angelo, Texas, USA; c Texas A&M Institute for Genome Sciences and Society, College of Veterinary Medicine and Biomedical Sciences, Texas A&M University, College Station, Texas, USA; DOE Joint Genome Institute

## Abstract

This is a draft genome of an orf virus (ORFV) vaccine strain assembled via long- and short-read hybrid assembly. ORFV is a zoonotic pathogen that affects sheep and goats. The genome of the virus contained in the vaccine was found to have high similarity (98%) to those of other published strains.

## ANNOUNCEMENT

Contagious ecthyma is caused by the orf virus (ORFV), a member of the genus *Parapoxvirus* within the family *Poxviridae* that infects the skin and oral mucosa of ruminants, primarily sheep and goats, and causes painful vesicles, pustules, and proliferative lesions, typically around the mouth and nose (1, 2). The morbidity rate is approximately 89% in sheep, and the disease has a substantial economic impact (1, 3). The virus survives in scab material from ORFV lesions and can remain infectious for up to 15 years (4, 5).

A commercially available ovine ecthyma vaccine live virus (USDA product serial number 1821.51) was selected for sequencing because the virus is typically propagated in animals, and there is the potential for the vaccine strains to vary in sequence over time. Sequencing of the virus provides a reference point for future investigations if needed. The vaccine virus was used to inoculate ovine testicular cells (ATCC OA3.Ts) suspended in Dulbecco’s modified Eagle’s medium (Sigma-Aldrich). The infected cells were harvested after an additional 48 h of incubation by repeat freeze-thaw cycles alternating between −80°C and 25°C. DNA was extracted using a DNeasy blood and tissue kit (Qiagen) according to the manufacturer’s protocol. DNA quality was verified on a genomic DNA TapeStation (Agilent) prior to sequencing. Default parameters were used for all programs except where otherwise noted.

Illumina libraries were prepared using the Illumina Nextera DNA Flex library preparation kit following the manufacturer’s protocol and were sequenced with an Illumina MiSeq v3 kit (2 × 300 bp). All data were uploaded to the Illumina cloud-based resource BaseSpace for run monitoring, fastq file generation, demultiplexing, and adapter trimming. The sequencing output of paired-end read sets contained 1,725,292 reads of 301 bp. Reads under 200 bp were filtered and trimmed using Trimmomatic v0.39 (6), resulting in 1.4 million retained reads.

Nanopore libraries were prepared following the manufacturer’s protocol for 1D PCR barcoding of genomic DNA using the Nanopore SQK-RAD004 rapid sequencing kit. Sequencing data collection and base calling were performed by MinKNOW v3.4.8 software with real-time base calling enabled. Sequencing resulted in an output of read sets containing 1.6 Gbp of data. There were 956,309 Nanopore reads generated and 1,676,000,000 bp obtained from the library. Reads were corrected and trimmed using Canu v1.8 (7).

Ovine DNA Illumina reads were removed using the Texas A&M Institute for Genome Sciences and Society (TIGSS) Virus Identification Pipeline with Ovis aries ENSEMBL build 84. In brief, the paired-end reads were combined and mapped to the host genome assembly using Bowtie 2 v2.3.4.1 in local mapping mode (8, 9). The program next filtered out any bacterial reads by mapping to the GOTTCHA bacterial signature database at the species level (10), after which the final 31,626 filtered paired-end viral reads were deinterleaved and assembled using Unicycler v0.2.3 (11), resulting in a 10-contig assembly of 124,608 bp. The Nanopore reads were mapped to this assembly using minimap2 v2.14 (12). The mapped Nanopore reads (*n* = 27,573 reads) and the Illumina viral reads were assembled together, with a coverage of over 164×, using Unicycler in conservative mode to form a hybrid assembly of 7 contigs and 165,738 bases. This assembly was scaffolded to the complete ORFV reference genome OV-IA82 (GenBank accession number AY386263.1) using Ragout v2.1.1 (13, 14), resulting in a single scaffold of 2 contigs and 5 unplaced contigs. The unplaced contigs were confirmed by BLAST to be host contamination (15) and were subsequently discarded. The reference genome has 137,241 bp, compared to the genome described here, which has 134,882 bp, a difference of 2,359 bp.

The final draft genome consisted of a single scaffold containing 2 contigs with a total 134,882 bp and a GC content of 64.14%. The largest contig was 101,329 bp and the second largest was 33,553 bp long. The scaffold *N*_50_ value is 134,893 bp, and the contig *N*_50_ value is 101,329 bp. This assembly demonstrates high levels of identity to those of other published ORFV strains, including OV-IA82 (GenBank accession number AY386263.1; identity, 98.71%), B029 (KF837136.1; identity, 99.15%), OV-SA00 (AY386264.1; identity, 98.08%), and OV-HN3/12 (KY053526.1; identity, 98.37%), when compared using NCBI BLAST (16). Relative to the OV-IA82 sequence, there was a truncation at the beginning of the sequence, as the first base pair in accession number MN454854.1 paired with base pair 3313 of accession number AY386263.1.

### Data availability.

This genome sequence was deposited in GenBank under BioProject number PRJNA563624 and accession number MN454854. The raw reads are available under SRA accession numbers SRR10102957 (Nanopore fast5) and SRR10102956 (Illumina fastq). This announcement represents the first version of this genome.
